# Body mass index mediates the association between plasma lipid concentrations and the prevalence of obstructive sleep apnea among US adults: a cross-sectional study

**DOI:** 10.3389/fcvm.2024.1433884

**Published:** 2024-12-19

**Authors:** Xiao Hu, Jing Xu, Yang Gu

**Affiliations:** ^1^ Department of Cardiology, The Affiliated Huaian No.1 People’s Hospital of Nanjing Medical University, Huaian, Jiangsu, China; ^2^ Department of Respiratory and Critical Care Medicine, The Affiliated Huaian No.1 People’s Hospital of Nanjing Medical University, Huaian, Jiangsu, China

**Keywords:** obstructive sleep apnea, triglycerides, HDL-C, LDL-C, BMI

## Abstract

**Background:**

The association between obstructive sleep apnea (OSA) and plasma lipid concentrations is not consistent. This study aimed to investigate the association of plasma lipid concentrations with the prevalence of OSA among US adults, with an additional examination of the mediating effect of body mass index (BMI).

**Methods:**

This cross-sectional study included 8,086 individuals who participated in the National Health and Nutrition Examination Survey (NHANES), conducted from 2005 to 2008 and 2015–2018. Multivariable logistic regression analysis was conducted to compute the odds ratios (ORs) and 95% confidence intervals (CIs) for the association between plasma lipid concentrations and the prevalence of OSA. Additionally, subgroup analysis was used to explore the potential interactions. Generalized additive models (GAM) were constructed to evaluate the nonlinear relationships between lipid concentrations and OSA. Furthermore, mediation analysis was performed to assess the potential mediating role of BMI.

**Results:**

In the fully adjusted model, when comparing the lowest quartile, the ORs for the prevalence of OSA among participants in the highest quartile were 1.367 (95% CI, 1.107–1.688) for triglyceride and 1.212 (95% CI, 1.004–1.462) for low-density lipoprotein cholesterol (LDL-C). However, total cholesterol (TC) and high-density lipoprotein cholesterol (HDL-C) were not associated with OSA. Notably, the relationship between triglyceride and OSA differed in the subgroups of gender, race, and body mass index (BMI) (P for interaction <0.05). Furthermore, we discovered an inverted U-shaped association between triglyceride and OSA (inflection point: 0.813 mmol/L). Causal mediation analysis revealed that BMI significantly mediated the relationship between triglyceride and the prevalence of OSA.

**Conclusions:**

This study revealed that an elevated level of triglyceride increased the prevalence of OSA, and this effect was potentially mediated through BMI. Lowering triglyceride concentration may help to reduce the prevalence of OSA.

## Introduction

Obstructive sleep apnea (OSA) is a chronic intermittent sleep disorder characterized by recurrent apneas, sleep disruptions, and intermittent hypoxia (1). Although OSA is a breathing disorder that occurs during sleep, the relevant pathophysiological consequences of recurrent apnea tend not to regress upon awakening (2). The episode of apnea usually persists for a minute or less. When it remains stable night after night for years, there can be long-term consequences for patients with OSA. OSA leads to increased oxidative stress, sympathetic activity, and chronic low-grade inflammation that contribute to metabolic alterations such as dyslipidemia (3, 4), hypertension (5), diabetes (6), and cardiovascular diseases (7). Recent evidence suggests that individuals suffering from OSA are at an elevated risk for developing cardiovascular diseases (CVDs). A robust investigation indicates that nocturnal oxygen desaturation significantly contributes to the development of cardiovascular complications associated with OSA (8). This association is consequently related to higher mortality rates observed in patients with OSA (9).

Patients with OSA frequently suffer from dyslipidemia (10). An animal study showed that the alterations in lipid metabolism triggered by hypoxia were facilitated through the action of hepatic stearoyl coenzyme A desaturase 1 (11). This enzyme plays a crucial role in the biosynthesis of cholesterol esters and triglycerides, contributing to dyslipidemia by enhancing the secretion of lipoproteins (12). Another cohort study described the association of severe OSA with increased triglyceride concentrations and decreased high-density lipoprotein cholesterol (HDL-C) concentrations (13). Intermittent hypoxemia may disrupt lipid metabolism by enhancing the breakdown of adipose tissue, leading to an increased flow of free fatty acids to the liver. This process occurs through the upregulation of hepatic triglyceride biosynthesis and the secretion of lipoproteins, while simultaneously suppressing the clearance of these lipoproteins from the bloodstream (14). However, other systematic reviews have provided uncertain evidence to support a direct association between lipid concentration and OSA (15, 16).

The causal relationship between lipid profiles and OSA requires further investigation due to several challenges. One significant issue is the potential confounding by other factors. Obesity has been established as a factor that affects lipid profiles (17), and it is also acknowledged as a significant contributor to OSA (18). This dual association complicates the understanding of whether the observed relationship between lipid profiles and OSA is indeed causal or if it comes from their shared connection with obesity. Thus, unravelling the interrelated factor is essential for clarifying the nature of the relationship between lipid profiles and OSA. Limited research has been undertaken regarding this relationship, and its nature remains to be fully clarified. Therefore, this research endeavors to address a significant gap in the current understanding of the interplay between OSA and lipid metabolism and obesity. By elucidating the lipidomic changes associated with OSA, it is anticipated that the study will provide valuable insights that can enhance diagnostic and therapeutic strategies, ultimately improving outcomes for individuals affected by this prevalent sleep disorder.

## Materials and methods

### Study population

NHANES is a continuous, cross-sectional health and nutrition survey conducted by selecting representative non-institutionalized American residents. Data are publicly available and can be obtained at https://www.cdc.gov/nchs/nhanes. In this study, we extracted and integrated data from the NHANES 2005–2008 and 2015–2018 circles, which include a more comprehensive questionnaire about sleep disorders than any other cycles. Our initial data sample comprises 22,202 individuals aged 20 years or older. After excluding participants with missing data for plasma lipid concentrations and OSA symptoms, 8,086 participants (3,950 men and 4,136 women) were finally included in our analysis.

### Identification of OSA

The definition of OSA symptoms is based on the answers to three dichotomous questions (19). These include the following: (1) Snoring three nights or more per week; (2) gasping, snorting, or stopping breathing three nights or more per week; or (3) Feeling overly sleepy during the daytime 16–30 times per month. Participants who responded yes to at least two of the three questions were considered positive for having OSA symptoms.

### Plasma lipid concentration measurements

Serum levels of triglyceride, HDL-C, TC (Total cholesterol), and LDL-C (low-density lipoprotein cholesterol) for each specimen were measured using Roche Cobas 6,000 and Roche modular P chemistry analyzers.

### Covariates

According to previous findings, confounders with theoretically plausible explanations were selected as covariates. Self-reported demographic data included age, gender, race (Mexican-American, other Hispanic, non-Hispanic White, non-Hispanic Black, and other race), income, and education. The poverty income ratio (PIR) referred to the ratio of household income to poverty threshold income (20). As indicators of socioeconomic status, PIR was classified as <1.3, 1.3–1.8, and >1.8 (21). Education level was categorized as less than high school, high school or equivalent, and greater than high school (22). We classified BMI (weight/height^2^) into three categories: normal/underweight (BMI < 25 kg/m^2^), overweight (BMI between 25 and 30 kg/m^2^), and obese (BMI ≥ 30 kg/m^2^) (23). Alcohol consumption was categorized according to the National Institute on Alcohol Abuse and Alcoholism (NIAAA) criteria in the National Institute of Health, with binge drinking defined as ≥ 5 drinks/day for men or ≥4 drinks/day for women, heavy drinking defined as 3–4 drinks day for men or 2–3 drinks day for women, moderate drinking defined as 1–2 drinks/day for men or 1 drink/day for women, and none (24). Smoking status was categorized as current, prior, or never (25). Physical activity was determined by whether the participants were walking or bicycling (26). Individuals with a mean systolic blood pressure of at least 140 mmHg, a mean diastolic blood pressure of at least 90 mmHg, or current use of antihypertensive agents were defined as having hypertension (27). Cardiovascular events were defined by self-reported history of cardiovascular diseases (CVDs). The outcome was a composite of heart failure (HF), coronary heart disease (CHD), myocardial infarction (MI), angina pectoris, or stroke (28). Participants who responded with “yes” to any of the following questions were defined as CVDs: “Has a doctor or other health professional ever told you that you had a heart attack (also called myocardial infarction) coronary heart disease congestive heart failure angina (also called angina pectoris) a stroke?”. The hepatic steatosis index (HSI) was applied to assess the extent of steatosis in individuals with non-alcoholic fatty liver disease (NAFLD). The formula for calculating HSI is represented as follows: HSI = 8 × (ALT/AST) + BMI (+2 for individuals with diabetes and +2 for females). A standard threshold value of 36 for HSI is utilized for the diagnosis of NAFLD (29). Use of lipid-lowering drugs was obtained from the Drug Questionnaire. Laboratory analyses were conducted, including the assessment of alanine aminotransferase (ALT), serum albumin (ALB), blood urea nitrogen (BUN), and serum creatinine (Scr) at baseline.

### Statistical analyses

Data were recorded using categorical or continuous variables. Distributed continuous variables were expressed as weighted mean ± standard deviations (mean ± SD). For categorical variables, proportions and weighted percentages with corresponding 95% confidence intervals (CIs) were computed. Categorical data were compared using the chi-square test, while continuous data were compared using Student's *t*-test. Multivariable logistic regression analysis was conducted to assess the association between plasma lipid concentrations and OSA. Odds ratio (OR) was used to determine the strength of the association. Multicollinearity was evaluated by calculating the variance inflation factors (VIF). Only those with VIF < 10 was included in the model. *P*-value of Goodness-of-fit test was >0.05, indicating that the established model fits well. The confounding factor was selected based on clinically relevance or when added it to this model, changed the matched odds ratio on the outcome measure by at least 10% or it was significantly associated with OSA. Furthermore, to ensure parsimony of the final logistic regression model, variables for inclusion were determined based on the number of events available. Model 1 was the unadjusted model. Model 2 was adjusted for demographic characteristics (age, gender, and race). Model 3 included adjustment for demographic information (age, gender, and race), individual characteristics (PIR, BMI, and level of education), health behaviors (daily alcohol consumption, physical activity status, and smoking status), comorbid conditions (history of diabetes, hypertension, CVDs, and NAFLD), laboratory tests (ALT, ALB, and Scr), and medication history (use of lipid-lowering agents). Given the significant health implications associated with plasma lipids, it is essential to investigate the various factors that affect them. Non-genetic determinants, such as socio-demographic variables and lifestyle choices—including age, gender, alcohol consumption, smoking, physical activity, BMI, comorbidities, and medication regimens—play a crucial role. Lifestyle factors such as physical activity, alcohol consumption, smoking, and BMI are modifiable and can be effectively managed to regulate lipid levels. Therefore, we reported stratified results of triglyceride, LDL-C, and HDL-C by age (<60 or ≥60 years), gender (male or female), race (non-Hispanic White or other), BMI (<30 or ≥30), smoking status (never, ever or current), alcohol consumption (nondrinker or drinker), physically active (inactive or active), hypertension (yes or no), diabetes (yes or no), NAFLD(yes or no), and use of lipid-lowering agents (yes or no). Potential interactions were performed using the likelihood ratio test. To further assess the nonlinear relationships, generalized additive models (GAM) were employed. *P*-values for the log-likelihood ratio test were used to compare the linear regression model with a two-piecewise linear regression model. Nonlinearity was indicated when the *P*-value for the log-likelihood ratio was less than 0.05. Subsequently, we determined the inflection point to develop a two-piecewise linear regression model using a recursive algorithm. The corresponding threshold level was estimated based on the maximum likelihood method. Causal mediation analyses were conducted to investigate whether BMI mediates the associations of plasma lipid concentrations with OSA. Mediation analyses could quantify the total effect (association between plasma lipid concentrations and OSA), natural direct effect (total effect without the influence of BMI), and natural indirect effect (effect of plasma lipid concentrations on OSA attributed to BMI). All statistical analyses were performed by R version 4.2.3 (R Foundation for Statistical Computing, Vienna), STATA 16.0 (Stata Corp, College Station, TX, USA), and Empower stats (http://www.empowerstats.com, X&Y Solutions, Inc, CA, USA).

## Results

### Baseline characteristics of the study population

Baseline characteristics of the 8,086 study participants stratified by OSA are presented in Table 1 based on weighted analyses. Compared to participants without OSA, those with OSA were generally older, predominantly male, and more likely to be obese. Additionally, they had a higher likelihood of being current smokers and had a history of conditions such as diabetes, hypertension, CVDs, and NAFLD. These individuals were also more likely to be receiving lipid-lowering treatment. Furthermore, they also had higher levels of ALT, BUN, Scr, triglyceride, TC, and LDL-C, while showing decreased levels of ALB and HDL-C.

**Table 1:** Weighted characteristics of participants stratified by OSA, NHANES 2005–2008 and 2015–2018.

	Total (*n* = 8,086)	Non-OSA (*n* = 6,935)	OSA (*n* = 1,151)	*P*-value
Age (year)	47.31 ± 16.77	46.86 ± 16.95	50.15 ± 15.31	<0.001
Gender (%)				<0.001
Male	48.74 (47.25–50.24)	47.14 (45.52–48.76)	58.74 (54.86–62.51)	
Female	51.26 (49.76–52.75)	52.86 (51.24–54.48)	41.26 (37.49–45.14)	
Race (%)				0.487
Mexican American	8.45 (7.92–9.01)	8.62 (8.05–9.23)	7.34 (6.11–8.8)	
Other Hispanic	5.54 (5.08–6.04)	5.63 (5.13–6.18)	4.99 (3.99–6.21)	
Non-Hispanic white	67.04 (65.83–68.23)	66.72 (65.4–68.01)	69.02 (65.93–71.96)	
Non-Hispanic black	10.77 (10.2–11.38)	10.8 (10.17–11.45)	10.64 (9.19–12.28)	
Other races	8.2 (7.51–8.95)	8.23 (7.47–9.05)	8.01 (6.41–9.97)	
Education (%)				<0.001
Less than high school	5.16 (4.74–5.63)	5.1 (4.65–5.61)	5.53 (4.45–6.85)	
High school or equivalent	34.55 (33.17–35.96)	33.49 (32.02–35)	41.15 (37.36–45.05)	
More than high school	60.27 (58.84–61.69)	61.39 (59.85–62.91)	53.3 (49.38–57.17)	
Not recorded	0.01 (0–0.03)	0.01 (0–0.02)	0.02 (0–0.17)	
Poverty-income ratio (%)				0.029
<1.3	17.2 (16.29–18.15)	16.99 (16.02–18.01)	18.52 (16.06–21.27)	
1.3–1.8	8.68 (8.02–9.38)	8.46 (7.77–9.21)	10.01 (8.11–12.28)	
≥1.8	67.27 (65.98–68.53)	67.44 (66.05–68.79)	66.22 (62.72–69.55)	
Not recorded	6.85 (6.19–7.59)	7.11 (6.37–7.93)	5.25 (3.97–6.92)	
BMI group (%)				<0.001
<25	30.18 (28.81–31.59)	32.59 (31.07–34.14)	15.18 (12.64–18.14)	
25–30	33.41 (32.02–34.83)	34.12 (32.6–35.66)	29.02 (25.56–32.75)	
≥30	36.41 (34.99–37.86)	33.3 (31.8–34.83)	55.8 (51.86–59.66)	
Drinking status (%)				<0.001
Non-drinkers	9.78 (8.99–10.62)	10.2 (9.33–11.13)	7.13 (5.6–9.05)	
Moderate-drinkers	32 (30.56–33.47)	32.26 (30.71–33.85)	30.34 (26.7–34.25)	
Heavy-drinkers	27.38 (26.06–28.74)	28.02 (26.58–29.5)	23.4 (20.22–26.92)	
Binge-drinkers	13.43 (12.43–14.49)	12.12 (11.09–13.23)	21.56 (18.49–24.99)	
Not recorded	17.42 (16.41–18.48)	17.4 (16.31–18.54)	17.55 (14.97–20.48)	
Smoking status (%)				<0.001
Never	54.41 (52.91–55.9)	56.09 (54.48–57.69)	43.9 (40.05–47.83)	
Prior	25.76 (24.44–27.14)	25.62 (24.19–27.1)	26.68 (23.31–30.35)	
Current	19.78 (18.65–20.96)	18.25 (17.08–19.48)	29.34 (25.89–33.04)	
Not recorded	0.05 (0.02–0.12)	0.04 (0.01–0.13)	0.08 (0.02–0.32)	
Physical activity level (%)				<0.001
Yes	22.08 (20.9–23.31)	22.83 (21.54–24.17)	17.45 (14.73–20.55)	
History of hypertension (%)				<0.001
Yes	36.64 (35.23–38.08)	34.72 (33.22–36.26)	48.62 (44.71–52.55)	
History of diabetes (%)				<0.001
Yes	9.51 (8.73–10.35)	8.78 (7.96–9.66)	14.07 (11.77–16.74)	
CVD (%)				<0.001
Yes	8.65 (7.94–9.42)	7.87 (7.13–8.67)	13.56 (11.34–16.12)	
NAFLD (%)				<0.001
Yes	55.19 (53.69–56.67)	52.32 (50.7–53.94)	73.03 (69.42–76.35)	
Lipid-lowering agents (%)				<0.001
Yes	22.11 (20.88–23.39)	20.28 (19.01–21.61)	33.48 (29.74–37.45)	
ALT, U/L	24.70 ± 17.56	24.32 ± 17.73	27.07 ± 16.22	<0.001
ALB, g/dl	4.20 ± 0.35	4.20 ± 0.35	4.15 ± 0.34	<0.001
BUN, mg/dl	13.62 ± 5.21	13.57 ± 5.15	13.93 ± 5.53	0.031
Scr, mg/dl	0.87 ± 0.32	0.87 ± 0.32	0.91 ± 0.33	<0.001
TC, mmol/L	4.97 ± 1.05	4.96 ± 1.04	5.03 ± 1.09	0.029
TG, mmol/L	1.32 ± 0.75	1.28 ± 0.73	1.55 ± 0.81	<0.001
HDL-C, mmol/L	1.43 ± 0.43	1.45 ± 0.44	1.30 ± 0.38	<0.001
LDL-C, mmol/L	2.94 ± 0.91	2.92 ± 0.91	3.02 ± 0.95	0.002

Continuous variables are described as weighted mean ± standard deviations (mean ± SD). Categorical variables are expressed as weighted percentages with corresponding 95% confidence intervals. *P*-values are weighted.

OSA, obstructive sleep apnea; BMI, body mass index; CVD, cardiovascular disease; NAFLD, non-alcoholic fatty liver disease; ALT, alanine aminotransferase; ALB, albumin; BUN, blood urea nitrogen; Scr, Serum creatinine; TC, total cholesterol; TG, triglycerides; HDL-C, high-density lipoprotein-cholesterol; LDL-C, low-density lipoprotein-cholesterol; NHANES, National Health and Nutrition Examination Survey.

### Relationship between plasma lipid concentrations and the prevalence of OSA

To investigate the relationship between plasma lipid concentrations and the prevalence of OSA, three multivariable logistic regression models were constructed for analysis (Table 2). Compared to the lowest quartile in the non-adjusted model (model 1), individuals from the highest quartile of triglyceride and HDL-C had a 116% [OR, 2.162 (1.783, 2.621)] increased risk and a 55% [OR, 0.455 (0.377, 0.550)] decreased risk for the prevalence of OSA. Similar results were observed in Model 2. After adjusting for all confounding factors (model 3), compared with the lowest quartile group, the ORs with 95%CI for the prevalence of OSA were 1.277(1.037,1.572), 1.452(1.182,1.784), and 1.367(1.107,1.688) for triglyceride, and 1.180(0.976,1.426), 1.078(0.891,1.303), and 1.212(1.004,1.462) for LDL-C in the second, third, and highest quartile groups, respectively. The high levels of triglyceride and LDL-C (>75th percentile) had significant associations with the prevalence of OSA. However, TC and HDL-C were not associated with the prevalence of OSA.

**Table 2:** Associations between plasma lipid concentrations and OSA, NHANES 2005–2008 and 2015–2018.

	Model 1	Model 2	Model 3
	OR (95%CI) P for trend	OR (95%CI) P for trend	OR (95%CI) P for trend
**TC (mmol/L)**	0.964	0.864	0.093
Q1 (<4.19)	Reference	Reference	Reference
Q2 (4.19–4.86)	0.886 (0.741,1.060)	0.905 (0.756,1.084)	0.999 (0.827,1.206)
Q3 (4.86–5.61)	1.025 (0.861,1.220)	1.046 (0.877,1.247)	1.204 (0.999,1.451)
Q4 (≥5.61)	0.948 (0.794,1.131)	0.968 (0.809,1.158)	1.124 (0.930,1.360)
**TG (mmol/L)**	< 0.001	< 0.001	0.004
Q1 (<0.779)	Reference	Reference	Reference
Q2 (0.779–1.152)	1.613 (1.321,1.970) ***	1.531 (1.251,1.874) ***	1.277 (1.037,1.572) *
Q3 (1.152–1.694)	2.065 (1.701,2.506) ***	1.954 (1.603,2.382) ***	1.452 (1.182,1.784) ***
Q4 (≥1.694)	2.162 (1.783,2.621) ***	2.045 (1.676,2.495) ***	1.367 (1.107,1.688) **
**HDL-C (mmol/L)**	< 0.001	< 0.001	0.087
Q1 (<1.11)	Reference	Reference	Reference
Q2 (1.11–1.34)	0.858 (0.728,1.012)	0.874 (0.739,1.033)	1.034 (0.869,1.231)
Q3 (1.34–1.66)	0.655 (0.552,0.778) ***	0.692 (0.580,0.827) ***	0.977 (0.810,1.179)
Q4 (≥1.66)	0.455 (0.377,0.550) ***	0.484 (0.396,0.591) ***	0.839 (0.676,1.042)
**LDL-C (mmol/L)**	0.341	0.351	0.1
Q1 (<2.276)	Reference	Reference	Reference
Q2 (2.276–2.845)	1.040 (0.869,1.244)	1.064 (0.888,1.275)	1.180 (0.976,1.426)
Q3 (2.845–3.491)	1.002 (0.839,1.197)	1.013 (0.847,1.211)	1.078 (0.891,1.303)
Q4 (≥3.491)	1.106 (0.929,1.318)	1.110 (0.930,1.323)	1.212 (1.004,1.462) *

Abbreviations are the same as those in Table 1. Model 1: Non-adjusted model; Model 2 adjusted for age, gender, race; Model 3 adjusted for age, gender, race, education, poverty-income ratio, BMI, alcohol drinking status, smoking status, physical activity level, diabetes, hypertension, CVD, NAFLD, lipid-lowering agents, ALT, ALB, and Scr. *P < 0.05, **P < 0.01, ***P < 0.001. *P*-values were calculated by using Q1 as the reference. P for trend is presented as the differences between Q1, Q2, Q3 and Q4.

### Stratified analyses

We found significant interactions of triglyceride levels with gender, race, and BMI on the prevalence of OSA (Table 3). When stratified by gender (P-interaction = 0.039), female participants in the third and fourth quartile had a 52% [OR, 1.522 (1.151, 2.012)] and a 48% [OR, 1.48 (1.117, 1.962)] higher prevalence of OSA risk odds than those in the first quartile. Race also yielded a significant interaction effect (P-interaction = 0.047). Compared with the first triglyceride quartile group, the prevalence of OSA from other races in the third and fourth quartile indicated high risk odds (OR, 1.635 [1.264, 2.116], OR, 1.435 [1.095, 1.880). However, this phenomenon was not noted in non-Hispanic White individuals. In patients with BMI ≥ 30, the relationship between triglyceride and the prevalence of OSA was more prominent (P-interaction = 0.043). The fourth quartile group had a 92% higher risk odd than the first quartile group, with an OR of 1.922 (1.389, 2.660). However, no significant interactions were found between LDL-C and HDL-C and stratified variables (Supplementary Table S1).

**Table 3:** Associations of TG and LDL-C With the prevalence of OSA in Various subgroups, NHANES 2005–2008 and 2015–2018.

Characteristic	Odds ratio (95% CIs) by quartile				*P*-value for interaction
	Quartile 1	Quartile 2	Quartile 3	Quartile 4	
TG (mmol/L)	<0.779	0.779–1.152	1.152–1.694	≥1.694	
Age (year)					0.784
<60	Reference	1.387 (1.070,1.798) *	1.538 (1.188,1.991) **	1.404 (1.078,1.830) *	
≥60	Reference	1.102 (0.774,1.569)	1.300 (0.916,1.845)	1.178 (0.820,1.694)	
Gender					0.039
Male	Reference	1.539 (1.138,2.081) **	1.338 (0.953,1.964)	1.251 (0.900,1.739)	
Female	Reference	1.108 (0.829,1.481)	1.522 (1.151,2.012) **	1.480 (1.117,1.962) **	
Race					0.047
Non-Hispanic White	Reference	1.397 (0.998,1.957)	1.368 (0.977,1.915)	1.344 (0.971,2.012)	
Other	Reference	1.251 (0.959,1.631)	1.635 (1.264,2.116) ***	1.435 (1.095,1.880) **	
BMI (kg/m^2^)					0.043
<30	Reference	1.141 (0.872,1.494)	1.326 (1.012,1.738) *	1.059 (0.791,1.418)	
≥30	Reference	1.640 (1.176,2.288) **	1.870 (1.351,2.587) ***	1.922 (1.389,2.660) ***	
Smoking status					0.136
Never	Reference	1.580 (1.177,2.122) **	1.570 (1.162,2.121) **	1.346 (0.981,1.848)	
Ever or current	Reference	1.114 (0.827,1.501)	1.473 (1.106,1.962) **	1.436 (1.075,1.920) *	
Alcohol consumption					0.324
Nondrinker	Reference	1.452 (0.734,2.873)	1.036 (0.496,2.164)	1.562 (0.766,3.185)	
Drinker	Reference	1.237 (0.966,1.582)	1.468 (1.152,1.870) **	1.281 (0.997,1.647)	
Physical activity					0.970
Inactive	Reference	1.321 (1.046,1.669) *	1.480 (1.174,1.865) **	1.382 (1.090,1.752) **	
Active	Reference	1.169 (0.734,1.860)	1.412 (0.887,2.247)	1.321 (0.823,2.122)	
Diabetes					0.206
Yes	Reference	0.837 (0.482,1.455)	1.141 (0.667,1.953)	1.271 (0.749,2.156)	
No	Reference	1.395 (1.108,1.757) **	1.552 (1.233,1.953) ***	1.385 (1.089,1.760) **	
Hypertension					0.831
Yes	Reference	1.396 (1.027,1.899) *	1.532 (1.132,2.074) **	1.370 (1.005,1.867) *	
No	Reference	1.211 (0.902,1.626)	1.443 (1.078,1.931) *	1.393 (1.033,1.878) *	
NAFLD					0.783
Yes	Reference	1.385 (1.050,1.825) *	1.501 (1.147,1.965) **	1.458 (1.113,1.909) **	
No	Reference	1.169 (0.845,1.618)	1.458 (1.049,2.026) *	1.227 (0.848,1.777)	
Lipid-lowering agents					0.613
Yes	Reference	1.109 (0.737,1.669)	1.180 (0.789,1.763)	1.209 (0.881,1.801)	
No	Reference	1.344 (1.053,1.715) *	1.570 (1.232,1.999) ***	1.351 (1.049,1.741) *	
LDL-C (mmol/L)	<2.276	2.276–2.845	2.845–3.491	≥3.491	
Age (year)					0.801
<60	Reference	1.114 (0.868,1.429)	1.064 (0.833,1.359)	1.099 (0.862,1.401)	
≥60	Reference	1.202 (0.894,1.616)	1.041 (0.762,1.422)	1.286 (0.940,1.760)	
Gender					0.151
Male	Reference	1.281 (0.994,1.651)	1.073 (0.828,1.389)	1.389 (1.076,1.793) *	
Female	Reference	1.041 (0.782,1.385)	1.097 (0.828,1.452)	0.996 (0.751,1.321)	
Race					0.571
Non-Hispanic White	Reference	1.043 (0.781,1.392)	1.119 (0.840,1.491)	1.175 (0.881,1.568)	
Other	Reference	1.274 (0.990,1.641)	1.055 (0.819,1.361)	1.232 (0.960,1.581)	
BMI (kg/m^2^)					0.428
<30	Reference	1.009 (0.772,1.320)	0.937 (0.713,1.231)	1.112 (0.854,1.446)	
≥30	Reference	1.347 (1.029,1.764) *	1.220 (0.935,1.592)	1.291 (0.985,1.691)	
Smoking status					0.985
Never	Reference	1.163 (0.873,1.549)	1.118 (0.845,1.480)	1.265 (0.958,1.670)	
Ever or current	Reference	1.157 (0.897,1.493)	1.078 (0.832,1.397)	1.181 (0.912,1.528)	
Alcohol consumption					0.889
Nondrinker	Reference	1.299 (0.689,2.448)	1.256 (0.659,2.394)	1.468 (0.776,2.815)	
Drinker	Reference	1.121 (0.891,1.410)	1.062 (0.846,1.333)	1.172 (0.934,1.471)	
Physical activity					0.956
Inactive	Reference	1.171 (0.949,1.446)	1.061 (0.859,1.312)	1.201 (0.974,1.482)	
Active	Reference	1.271 (0.821,1.968)	1.215 (0.786,1.878)	1.336 (0.869,2.054)	
Diabetes					0.661
Yes	Reference	1.285 (0.862,1.915)	0.934 (0.594,1.469)	1.295 (0.825,2.033)	
No	Reference	1.119 (0.893,1.403)	1.130 (0.907,1.408)	1.191 (0.956,1.482)	
Hypertension					0.917
Yes	Reference	1.153 (0.891,1.492)	1.115 (0.859,1.448)	1.187 (0.916,1.538)	
No	Reference	1.170 (0.876,1.562)	0.997 (0.748,1.328)	1.156 (0.871,1.534)	
NAFLD					0.416
Yes	Reference	1.261 (0.999,1.592)	1.163 (0.924,1.464)	1.305 (1.038,1.640) *	
No	Reference	0.958 (0.689,1.333)	0.875 (0.621,1.233)	0.961 (0.685,1.348)	
Lipid-lowering agents					0.518
Yes	Reference	1.229 (0.888,1.702)	1.281 (0.914,1.795)	1.265 (0.908,1.761)	
No	Reference	1.092 (0.863,1.382)	0.937 (0.742,1.183)	1.061 (0.838,1.342)	

Abbreviations are the same as those in Table 1. Adjusted for age, gender, race, education, poverty-income ratio, BMI, alcohol drinking status, smoking status, physical activity level, diabetes, hypertension, CVD, NAFLD, lipid-lowering agents, ALT, ALB, and Scr. The strata variable was not included when stratifying by itself. *P < 0.05, **P < 0.01, ***P < 0.001. *P*-values were calculated by using Q1 as the reference.

### Detection of nonlinear relationships

**Figure 1:**
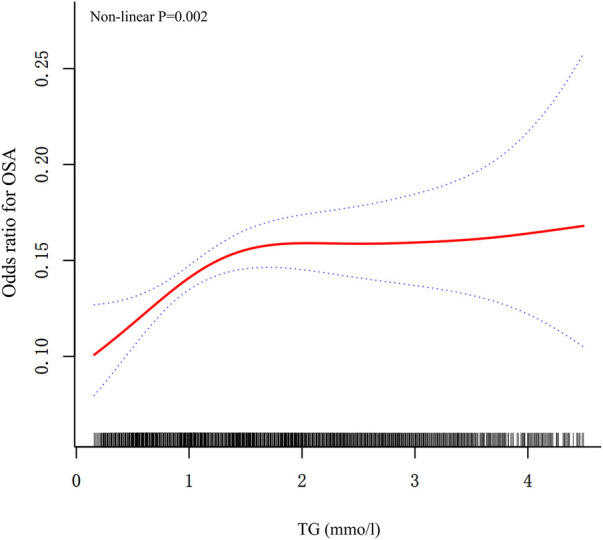
Plots of estimated smoothing spline functions with 95% confidence band for the generalized additive model between triglyceride and the prevalence of OSA. Analyses were adjusted for: age, gender, race, education, poverty-income ratio, BMI, alcohol drinking status, smoking status, physical activity level, diabetes, hypertension, CVD, NAFLD, lipid-lowering agents, ALT, ALB, and Scr. Solid lines represent trend lines and dotted lines represent 95% confidence intervals. OSA, obstructive sleep apnea; TG, triglyceride; BMI, body mass index; CVD, cardiovascular disease; NAFLD, non-alcoholic fatty liver disease; ALT, alanine aminotransferase; ALB, albumin; Scr, Serum creatinine.

By GAM, we discovered an inverted U-shaped association between triglyceride concentration and OSA (Figure 1), and the test for non-linearity was significant (*P* = 0.002 for log-likelihood ratio). When the triglyceride concentration was less than the turning point (0.813 mmol/L), per unit increase in the triglyceride concentration was correlated with a 249% greater adjusted OR of developing OSA [OR, 3.493 (1.663, 7.334); Table 4]. When serum triglyceride concentration exceeded the inflection point, there was no association between the triglyceride and the prevalence of OSA [OR, 1.051 (0.955, 1.555)]. Furthermore, the LDL-C level was not associated with the prevalence of OSA when it was entered in the analysis as a continuous variable (Table 4).

**Table 4:** Threshold effect analysis of TG and LDL-C on the prevalence of OSA.

Adjusted OR (95% CI), *P*-value	
TG (mmol/L)	
Fitting by the standard linear model	1.119 (1.028, 1.219) 0.010
Fitting by the two-piecewise linear mode, inflection point	0.813 mmol/L
TG < 0.813 mmol/L	3.493 (1.663, 7.334) 0.001
TG ≥ 0.813 mmol/L	1.051 (0.955, 1.155) 0.309
P for Log-likelihood ratio	0.002
LDL-C(mmol/L)	
Fitting by the standard linear model	1.059 (0.989, 1.135) 0.102
Fitting by the two-piecewise linear mode, inflection point	2.586 mmol/L
LDL-C < 2.586 mmol/L	1.165 (0.950, 1.428) 0.144
LDL-C ≥ 2.586 mmol/L	1.023 (0.926, 1.130) 0.654
P for Log-likelihood ratio	0.330

Abbreviations are the same as those in Table1. Adjusted for age, gender, race, education, poverty-income ratio, BMI, alcohol drinking status, smoking status, physical activity level, diabetes, hypertension, CVD, NAFLD, lipid-lowering agents, ALT, ALB, and Scr.

### Mediation analysis

**Figure 2:**
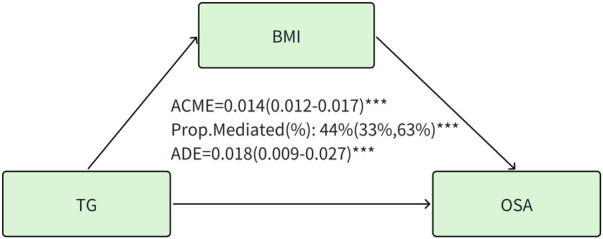
Mediation effect of BMI in the association of triglyceride with the prevalence of OSA. TG, triglyceride. ACME, average causal mediation effects (indirect effect); ADE, average direct effects. BMI, body mass index. *** *P* < 0.001.

A significant mediation effect of BMI was observed in the association of triglyceride with the prevalence of OSA. The mediated proportion (%) of BMI in the relationship of triglyceride with OSA was 44% (95%CI: 33%, 63%) (Figure 2). No mediating effects of BMI were observed in HDL, LDL, and TC.

## Discussion

This study examined the relationship between serum lipid concentrations and the prevalence of OSA in a large-scale U.S. general population. We discovered a significant association between triglyceride and the prevalence of OSA, as illustrated by an inverted U-shaped pattern. Significant interactions of triglyceride with gender, race, and BMI on the prevalence of OSA were also observed. The high level of LDL-C (>75th percentile) also had a significant association with the prevalence of OSA. Nevertheless, this study did not show that TC and HDL-C were correlated with OSA. Furthermore, causal mediation analysis demonstrated a significant mediation effect of BMI in the association of triglyceride with the prevalence of OSA, with the mediated proportion of 44%.

### Stratified analyses of lipid concentrations with OSA

We also performed stratified analyses of the effects of serum lipid concentrations on the prevalence of OSA in several categories, and the findings showed that the results differed among groups. A significant interaction between gender and triglyceride levels was observed on the odds of developing OSA. Our study established an unexpected association between triglyceride and females, but not males. The differentiation of body composition between sexes predominantly occurs during the stages of puberty and is influenced by the effects of sex hormones (30). Generally, females exhibit a greater overall fat content in their bodies, which is associated with insulin resistance (31). An increase in body fat percentage has been linked to an elevation in plasma triglyceride levels (32). We hypothesize that elevated overall adiposity could diminish the subcutaneous adipose tissue's ability to accommodate additional fat, resulting in an increased accumulation of fat in visceral and other ectopic locations. This metabolically active visceral fat is known to secrete various inflammatory cytokines and release free fatty acids into the portal circulation, which may subsequently disrupt hepatic metabolism. This disruption could lead to diminished hepatic insulin clearance, heightened synthesis of triglyceride-rich lipoproteins, and augmented hepatic glucose production (33). Furthermore, an increase in overall adiposity also leads to a larger reserve of abdominal subcutaneous fat, which exhibits significant lipolytic activity, thereby increasing the flow of free fatty acids and further contributing to insulin resistance and the risk of cardiovascular disease (34). Studies have reported that increased triglyceride levels are associated with high CVD risks in specific subgroups such as women (35–38). Meanwhile, hypertriglyceridemia was not correlated with CVD risks in men (37). A meta-analysis of prospective studies has also suggested that for per 1 mmol/L increase in triglyceride concentrations, the risk of CVD increases by 37% in women and 12% in men regardless of other risk factors (38). OSA and CVD are closely linked, as evidenced by accumulating findings. Further investigations of gender-specific pathways that affect triglyceride are needed to understand the prevalence of OSA.

Race also yielded a significant interaction effect on triglyceride. Compared with the first triglyceride quartile group, the prevalence of OSA increased 1.635 times with the 3rd and 1.435 times with the 4th quartile group in other races. However, this phenomenon was not observed in non-Hispanic White individuals, which indicates that various races have distinct effects on OSA. Racial differences in triglyceride concentrations may be influenced by a combination of lifestyle, genetic, and cultural factors (39). It is widely recognized that Black and Hispanic Americans experience lower rates of insurance coverage and face challenges in accessing healthcare, which is particularly evident in their increased dependence on emergency departments for medical services (40). Therefore, Black and Hispanic Americans had prevalence of dyslipidemia that was comparable to that of non-Hispanic whites but were less likely to be treated and controlled (41). A previous study also showed that African Americans have a higher prevalence of OSA than white patients (42).

Women generally exhibit higher levels of HDL compared to men, which can be attributed to the effects of estrogen and testosterone on hepatic lipase activity. Hepatic lipase is crucial in HDL metabolism, and its levels are inversely related to HDL levels (43). Specifically, estrogen tends to lower hepatic lipase levels, while testosterone tends to raise them. The relationship between age and HDL levels has been demonstrated in various studies. One study indicates that older African American women tend to have higher HDL levels (44). However, in the current study, no significant interactions were observed between HDL-C and the stratified variables identified in these previous studies. This suggests that further research with larger sample sizes is necessary to better understand the mechanisms that contribute to the variations in HDL levels.

### Nonlinear relationships between lipid concentration and OSA

Our study revealed that elevated triglyceride was significantly associated with an increased prevalence of OSA. Furthermore, the GAM model illustrated a nonlinear pattern, with a critical threshold identified at a value of 0.813. However, HDL, LDL, and TC had no causal effects on OSA. There are several potential interpretations. Triglyceride is characterized as hydrophobic, non-polar, neutral compounds. This structural composition renders triglyceride insoluble in water, facilitating their aggregation and subsequent deposition within extracellular fluid. Elevated blood triglyceride levels lead to an accumulation of triglyceride in different areas of the body. Oral and pharyngeal triglyceride accumulations can directly narrow the airway (45, 46). Patients can compensate for narrowing of the upper airway by enhancing the activity of the upper airway muscles while they are awake. However, this protective mechanism diminishes during sleep due to muscle relaxation (45). A study conducted by Tang et al. (47) indicated that triglyceride might have a causal role in the development of OSA. This is attributed to the hydrophobic nature of triglyceride, which leads to their aggregation and accumulation in extracellular fluids. In contrast, HDL and TC possess hydrophilic polar functional groups. The hydrophilic properties of these substances make them soluble in the saline-rich environment of the body (48). As a result, even when present in high concentrations, they are less likely to deposit and accumulate in areas such as the oral cavity, pharynx, neck, and abdomen, which could potentially block the airway. Additionally, these substances do not have the capacity to form adipose tissue, a key factor that can lead to metabolic dysregulation, as seen with triglycerides. Therefore, it can be concluded that the hydrophilic nature of these compounds suggests they do not have a significant direct causal link to OSA, like triglyceride. In addition, LDL and HDL primarily function to transport TC rather than triglyceride. Variations in the levels of LDL and HDL directly affect TC levels, while having minimal impact on triglyceride. TC, which is the main lipid component of both LDL and HDL, plays a crucial role as a structural element of cell membranes, helping to maintain proper membrane permeability and fluidity (49). Additionally, TC is vital for the synthesis of steroid hormones, bile acids, and vitamin D (50). Its hydrophilic group (-OH) makes TC less prone to accumulation compared to triglyceride. Consequently, the physiological roles of HDL, TC, and LDL indicate that they are unlikely to contribute to OSA indirectly through triglyceride pathways.

The observed nonlinear relationship between triglyceride and the prevalence of OSA could be attributed to the fact that individuals without dyslipidemia might demonstrate an increased vulnerability to the accumulation of visceral fat. This accumulation of visceral fat may subsequently elevate their risk of developing OSA in comparison to individuals with dyslipidemia (51). Interestingly, Deng H et al. (51) demonstrated that among participants without dyslipidemia, parameters such as body fat percentage, waist-to-hip ratio, lipid accumulation product, and resting metabolic rate exhibited a stronger correlation with the occurrence of OSA compared to those with dyslipidemia. Another explanation is that these patients were receiving lipid-lowering treatment, which may explain the relatively low triglyceride concentrations noted in quartile 1 and quartile 2.

So far, studies conducted on the evaluation of lipid status and sleep have mainly adopted OSA and lipid abnormalities through logistic/linear regression, which might result in potential residual confounding (52, 53). By contrast, our study reported a solid non-linear association between triglyceride and OSA in a large sample of participants. A strength of our study was the application of GAM in detecting the nonlinear curved-shape correlation. Several studies have demonstrated GAM could improve the risk adjustment compared to linear models (54, 55). Our results may provide a plausible theoretical foundation for future research in OSA prevention.

### BMI mediates the lipid concentration and OSA

The result of mediation analysis suggested that part of the effect of the triglyceride on OSA was realized through its effect on BMI. Furthermore, in the subgroup analyses regarding the association between triglyceride concentration and OSA, the strength of this relationship was found to be more pronounced in participants who were obese (*P* for interaction = 0.043). Obesity is a significant contributor to the development of OSA, which has been validated by numerous studies (56, 57). A review highlighted that obesity was a significant factor contributing to the increased prevalence of hypertriglyceridemia (58). Consequently, we evaluated how obesity influences the causal relationship between triglyceride levels and the susceptibility to OSA. We demonstrated that the causal impact of triglyceride on the prevalence of OSA was significant with the confounding effects of obesity. Abdominal fat accumulation, which includes both subcutaneous fat and visceral fat surrounding the organs in the abdominal cavity, significantly raises abdominal pressure. This increase in pressure can lead to a reduction in lung volume. When lung volume decreases, it may result in diminished longitudinal tracheal traction forces and reduced tension in the pharyngeal walls. These changes can predispose individuals to airway narrowing, potentially impacting respiratory function (59). Neck fat accumulation contributes to the increased collapsibility of the upper airway, which in turn reduces the effectiveness of the dilator muscles during contraction (60). This process can lead to sarcopenia, denervation, and dysfunction of skeletal muscles. Such alterations are associated with upper airway narrowing and the development of OSA. Classification of BMI may give more reliable evidence and provide novel targets for the prevention of obesity-related OSA.

As a clinical implication of our findings, physicians should pay more attention to the association between dyslipidemia and OSA. Subgroup analysis also showed that the prevalence of OSA is markedly greater among individuals who have not undergone lipid-lowering therapy when compared with those who have received such treatment. This observation indicates that lipid-lowering therapy plays a crucial role in diminishing the prevalence of OSA. Physicians can enhance the identification of individuals with elevated triglyceride levels for OSA screening, as triglyceride serves as an effective diagnostic biomarker. Additionally, clinicians can create more tailored treatment plans for patients by utilizing triglyceride levels as a basis for their therapeutic strategies.

### Limitations

Our study has several limitations to consider: (1) We could only identify some typical symptoms of OSA via the sleep questionnaire, such as snoring and daytime sleep. The individuals were not assessed by home or laboratory sleep testing (61), so some of the respondents with OSA might go undiagnosed. Using the STOP-Bang questionnaire may be a good supplementary method for screening suspected patients with OSA in the future (62). (2) Relying solely on data from US adults may restrict the applicability of the findings to other populations. (3) The cross-sectional design limits the ability to observe changes over time in physical indicators. Further researches are needed to elucidate the underlying mechanism of interaction on the complex balance between OSA symptoms and plasma lipid concentration.

## Conclusion

In conclusion, this study using NHANES data shows a significant relationship between triglyceride levels and the prevalence of OSA, indicating that its potential as a predictive indicator for OSA. These association was particularly prominent in female individuals, individuals from other races, and those who were obese. Causal mediation analysis demonstrated a significant mediation effect of BMI in the association of triglyceride with OSA. Maintaining a normal or low BMI could be a valuable recommendation for decreasing the prevalence of OSA in the adult population.

## Data Availability

The raw data supporting the conclusions of this article will be made available by the authors, without undue reservation.
