# Supplementary material for: Body mass index mediates the association between plasma lipid concentrations and the prevalence of obstructive sleep apnea among US adults: a cross-sectional study
Source: Front Cardiovasc Med. 2024 Dec 19;11:1433884. doi: 10.3389/fcvm.2024.1433884 (PMC11693669; doi:10.3389/fcvm.2024.1433884)
Supplement: Supplementary file 1 [file Table1.docx]

**Table S1.** Associations of HDL-C With Prevalence of OSA in Various Subgroups, NHANES 2005–2008 and 2015–2018.

| **Characteristic** | **Odds ratio (95% CIs) by quartile** | | | | **P value for interaction** |
| --- | --- | --- | --- | --- | --- |
|  | **Quartile 1** | **Quartile 2** | **Quartile 3** | **Quartile 4** |  |
| **HDL-C (mmol/l)** | <1.11 | 1.11-1.34 | 1.34-1.66 | ≥1.66 |  |
| Age (year) |  |  |  |  | 0.447 |
| <60 | Reference | 0.991(0.797,1.233) | 1.002(0.793,1.265) | 0.780(0.590,1.030) |  |
| ≥60 | Reference | 1.254(0.933,1.685) | 1.043(0.756,1.438) | 1.021(0.718,1.451) |  |
| Gender |  |  |  |  | 0.937 |
| Male | Reference | 1.028(0.832,1.270) | 0.965(0.754,1.234) | 0.864(0.631,1.181) |  |
| Female | Reference | 1.055(0.772,1.443) | 0.966(0.709,1.317) | 0.769(0.554,1.068) |  |
| Race |  |  |  |  | 0.931 |
| Non-Hispanic White | Reference | 1.022(0.787,1.326) | 0.898(0.675,1.194) | 0.818(0.591,1.131) |  |
| Other | Reference | 1.016(0.805,1.283) | 0.963(0.752,1.232) | 0.763(0.575,1.013) |  |
| BMI (kg/m^2^) |  |  |  |  | 0.197 |
| <30 | Reference | 0.937(0.711,1.235) | 1.079(0.822,1.416) | 0.829(0.616,1.115) |  |
| ≥30 | Reference | 1.120(0.893,1.404) | 0.863(0.661,1.127) | 0.768(0.552,1.070) |  |
| Smoking status |  |  |  |  | 0.826 |
| Never | Reference | 1.017(0.781,1.326) | 0.968 (0.735,1.275) | 0.732 (0.534,1.005) |  |
| Ever or current | Reference | 1.020(0.809,1.282) | 0.916(0.707,1.186) | 0.836(0.621,1.123) |  |
| Alcohol consumption |  |  |  |  | 0.138 |
| Nondrinker | Reference | 1.316(0.723,2.396) | 1.000(0.519,1.924) | 0.551(0.257,1.180) |  |
| Drinker | Reference | 0.962(0.778,1.189) | 0.973(0.777,1.219) | 0.930(0.721,1.201) |  |
| Physical activity |  |  |  |  | 0.310 |
| Inactive | Reference | 1.056(0.871,1.281) | 0.910(0.737,1.124) | 0.796(0.625,1.014) |  |
| Active | Reference | 0.974(0.651,1.457) | 1.281(0.839,1.956) | 0.895(0.549,1.461) |  |
| Diabetes |  |  |  |  | 0.127 |
| Yes | Reference | 1.206(0.822,1.769) | 0.692(0.434,1.103) | 0.842(0.495,1.433) |  |
| No | Reference | 0.971(0.793,1.188) | 1.006(0.814,1.243) | 0.766(0.599,0.979) |  |
| Hypertension |  |  |  |  | 0.44 |
| Yes | Reference | 1.069(0.840,1.362) | 0.939(0.718,1.227) | 0.961(0.714,1.293) |  |
| No | Reference | 1.080(0.835,1.398) | 0.997(0.759,1.311) | 0.731(0.526,1.017) |  |
| NAFLD |  |  |  |  | 0.849 |
| Yes | Reference | 1.013(0.830,1.236) | 0.979(0.783,1.225) | 1.858(0.653,1.126) |  |
| No | Reference | 1.123(0.780,1.617) | 0.982(0.682,1.415) | 0.783(0.532,1.153) |  |
| Lipid-lowering agents |  |  |  |  | 0.204 |
| Yes | Reference | 0.957(0.707,1.294) | 0.912(0.648,1.285) | 1.033(0.708,1.506) |  |
| No | Reference | 1.090(0.881,1.349) | 1.009(0.805,1.266) | 0.752(0.576,0.983) * |  |

Abbreviations are the same as those in Table1. Adjusted for age, gender, race, education, poverty-income ratio, BMI, alcohol drinking status, smoking status, physical activity level, diabetes, hypertension, CVD, NAFLD, lipid-lowering agents, ALT, ALB, and Scr. The strata variable was not included when stratifying by itself. *P<0.05. P-values were calculated by using Q1 as the reference.
